# Hypoxia-inducible factor-1α expression and breast cancer recurrence in a Danish population-based case control study

**DOI:** 10.1186/s13058-021-01480-1

**Published:** 2021-11-04

**Authors:** Lindsay J. Collin, Maret L. Maliniak, Deirdre P. Cronin-Fenton, Thomas P. Ahern, Kristina B. Christensen, Sinna P. Ulrichsen, Per Damkier, Stephen Hamilton-Dutoit, Rami Yacoub, Peer M. Christiansen, Henrik Toft Sørensen, Timothy L. Lash

**Affiliations:** 1 Department of Epidemiology, Rollins School of Public Health, Emory University, Atlanta, GA USA; 2 Department of Clinical Epidemiology, Aarhus University Hospital, Aarhus, Denmark; 3 Department of Population Health Sciences, Huntsman Cancer Institute, University of Utah, 2000 Circle of Hope Drive, Room 4746, Salt Lake City, UT 84112 USA; 4 Department of Surgery, The Robert Larner, M.D. College of Medicine, The University of Vermont, Burlington, VT USA; 5 Institute of Pathology, Aarhus University Hospital, Aarhus, Denmark; 6 Department of Clinical Biochemistry and Pharmacology, Odense University Hospital, Odense, Denmark; 7 Department of Clinical Research, University of Southern Denmark, Odense, Denmark; 8 Department of Plastic and Breast Surgery, Aarhus University Hospital, Aarhus, Denmark; 9 The Danish Breast Cancer Group, Aarhus, Denmark

**Keywords:** Breast cancer recurrence, Hypoxia-inducible factor 1, Tamoxifen resistance, Prognostic marker

## Abstract

**Background:**

Hypoxia-inducible factor-1α (HIF-1α) is a transcription factor that facilitates the adaptation of cancer cells to hypoxic conditions and may be prognostic of breast cancer recurrence. We evaluated the association of HIF-1α expression with breast cancer recurrence, and its association with timing of breast cancer recurrence.

**Methods:**

In this population-based case-control study, we included women diagnosed with stage I–III breast cancer between 1985 and 2001, aged 35–69 years, registered in the Danish Breast Cancer Group. We identified 541 cases of breast cancer recurrence among women with estrogen receptor (ER)-positive disease who were treated with tamoxifen for at least 1 year (ER+ TAM+). We also enrolled 300 breast cancer recurrence cases among women with ER-negative disease, not treated with tamoxifen, who survived at least 1 year (ER−/TAM−). Controls were recurrence-free breast cancer patients at the time of case diagnosis, matched to recurrence cases on ER/TAM status, date of surgery, menopausal status, cancer stage, and county of residence. Expression of HIF-1α was measured by immunohistochemistry on tissue microarrays. We fitted logistic regression models to compute odds ratios (ORs) and 95% confidence intervals (CIs) associating HIF-1α expression with recurrence, and with timing of recurrence.

**Results:**

HIF-1α expression was observed in 23% of cases and 20% of controls in the ER+/TAM+ stratum, and in 47% of cases and 48% of controls in the ER−/TAM− stratum. We observed a near-null association between HIF-1α expression in both ER/TAM groups (ER+/TAM+ OR = 1.21, 95%CI 0.88, 1.67 and ER−/TAM− OR = 0.97, 95%CI 0.68, 1.39). HIF-1α expression was not associated with time to recurrence among women in the ER+/TAM+ stratum, but was associated with early recurrence among women in the ER−/TAM− stratum.

**Conclusion:**

In this study, HIF-1α expression was not associated with breast cancer recurrence overall but may be associated with early recurrence among women diagnosed with ER− breast cancer.

**Supplementary Information:**

The online version contains supplementary material available at 10.1186/s13058-021-01480-1.

## Introduction

Nearly 90% of women diagnosed with breast cancer survive more than 10 years after their diagnosis [1]. Although targeted treatment protocols have contributed to improved survival, approximately 20–40% of breast cancer patients will have a recurrence by 20 years after their initial diagnosis [2, 3]. This substantial and prolonged risk of recurrence contributes to distress among breast cancer survivors [4]. Currently, the primary prognostic indicators for late recurrence include lymph node status and stage of breast cancer, which also predict early recurrence [2]. Novel biomarkers are needed to improve identification of patients who are at high risk of late recurrence, allowing for risk stratification of patients who may benefit from more intensive follow-up or prolonged treatment.

As tumors or metastases grow, cells in the tumor’s interior are more distant from blood supplies, leading to a hypoxic tumor microenvironment [5]. Tumor cells need to adapt to this hypoxic environment to facilitate tumor progression [6]. Hypoxia-inducible factor 1 (HIF-1α) is a transcription factor that facilitates the adaptation of cancer cells to hypoxic conditions [7], and may therefore serve as a prognostic marker for late recurrence [8]. Previous studies have shown that hypoxia-induced signaling enables tumor cells to survive during metabolic stress and to enter a prolonged quiescent state of tumor dormancy [9]. Additionally, a number of HIF target genes affect angiogenesis and proliferation of tumor cells, and the emergence from tumor dormancy to proliferative growth [10, 11]. HIF-1α expression is not detected in normal breast tissue, but is present in breast tumors, supporting its potential for use as a prognostic marker for cancer recurrence [7].

In this study, we evaluated the association between HIF-1α expression and breast cancer recurrence and its association with timing of breast cancer recurrence. In a set of exploratory analyses, we described differences in HIF-1α expression between primary and paired recurrent breast tumors, and evaluated if conservation of HIF-1α expression between primary and recurrent tumors was also associated with late recurrence.

## Materials and methods

### Study population

The study population and data collection have been described in detail elsewhere [12]. Briefly, the source population included stage I–III Danish female breast cancer patients, ages 35–69 years, diagnosed between 1985 and 2001, and registered with the Danish Breast Cancer Group (DBCG) [13]. Since 1977, the DBCG has enrolled nearly all Danish breast cancer patients younger than 70 years at diagnosis into its clinical database. Eligible patients were divided into two strata. The first stratum included patients whose tumors expressed estrogen receptor (ER) (≥ 10% of cells) and who were treated with tamoxifen (TAM) for at least one year (ER+/TAM+, *n* = 1826 patients). The second stratum included patients whose tumors did not express ER, who were not treated with TAM, and who survived at least one year (ER−/TAM−, *n* = 1808 patients). Patients not meeting these criteria were excluded (*n* = 7617 patients). Stratifying by ER and TAM status allowed separation of HIF-1α as predictive of TAM resistance, in which case an association would be observed in only the ER+/TAM+ stratum, and as a prognostic marker, in which case an association would be observed in both strata. Follow-up time was calculated from one year after breast cancer surgery until the first of (a) breast cancer recurrence, (b) death from any cause, (c) loss to follow-up, (d) completion of 10-year of follow-up, or (e) September 1, 2006.

Cases were defined as women with a diagnosis of a local, regional, or distant recurrence, or a contralateral breast cancer registered in the DBCG during follow-up [14]. Controls were selected from members of the source population who were not diagnosed with breast cancer recurrence nor with contralateral breast cancer at the follow-up time of the matched cases’ recurrence. Controls were matched to cases on ER/TAM group, menopausal status at diagnosis, date of breast cancer surgery (caliper matched+/− 12 months), county of residence, and Union for International Cancer Control (UICC) cancer stage at diagnosis. In the ER+/TAM+ stratum, 541 cases were identified; all were included in the analysis. In the ER−/TAM− stratum, 300 cases were identified, and frequency matched according to the distribution of stage and calendar period of diagnosis among the ER+/TAM+ case patients. Given the study design, the case-control odds ratio (OR) provides an unbiased estimate of the rate ratio for the association between HIF-1α expression and breast cancer recurrence rate [15].

### Data and tumor tissue collection

Every Danish citizen or legal resident is assigned a unique 10-digit Civil Personal Registration (CPR) number which allows unambiguous linkage across Danish registries [16]. We used the DBCG registry to obtain the following information: demographics (age, menopausal status, county of residence at diagnosis, and treating hospital), tumor characteristics (size, histology, histologic and nuclear grade, nodal involvement, ER status, and TNM stage), surgery type (mastectomy or breast conserving), radiation therapy, and receipt of chemotherapy and TAM therapy.

### Tissue microarray construction and immunohistochemistry

In Denmark, all paraffin blocks from pathological specimens are routinely archived after diagnosis. Patient CPR numbers were used to link patients in the study population to the Danish Pathology Data Bank, enabling us to locate and retrieve tumor blocks for 85% of study subjects [17]. For each case and control, formalin-fixed, paraffin-embedded (FFPE) primary tumor tissue blocks were retrieved from the pathology archives of treating hospitals. Paired FFPE blocks were collected when available for the local and distant recurrences of the 841 cases of breast cancer recurrence in the study sample. The purpose of collecting the recurrent tissues was to assay HIF-1α expression in the recurrent tumor, with the goal of comparing its expression at primary diagnosis with its expression at recurrence diagnosis. Laboratory personnel were blinded to all clinical information including case or control status, ER status, and receipt of TAM therapy.

**Fig. 1:**
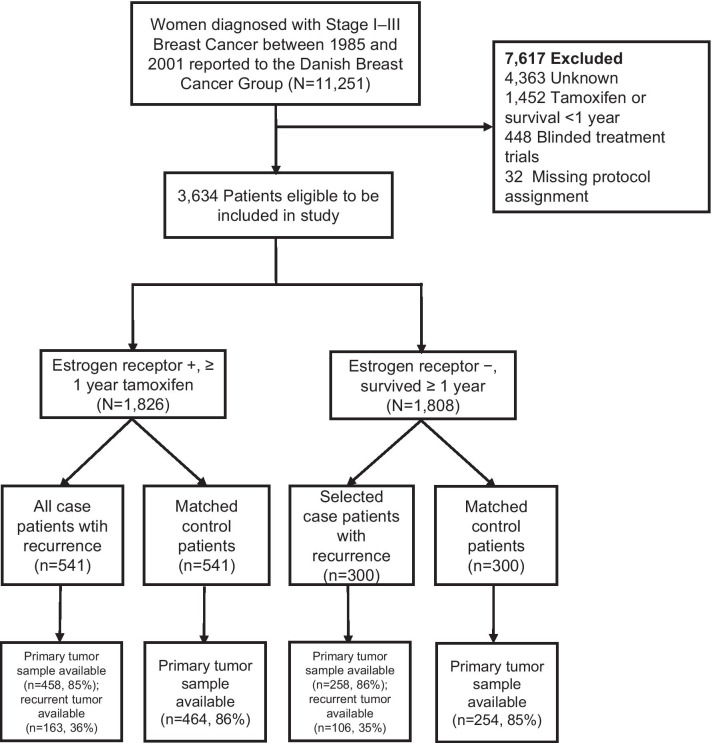
Population flow diagram for the selection of 1682 breast cancer patients diagnosed with stage I–III breast cancer and registered with the Danish Breast Cancer Group (1985–2001) by estrogen receptor (ER) status, and primary and recurrence tumor sample availability

Tissue microarrays (TMA) were constructed for the primary tumors (*n* = 1434, 85%) as well as the recurrent tumors (*n* = 269, 32%). Figure 1 illustrates the selection of study subjects to be used in the analysis. TMAs were constructed using standard techniques. A fresh section was cut from each study participant’s paraffin block and stained with hematoxylin and eosin. The diagnosis was confirmed by a study pathologist. Areas containing invasive breast carcinoma were identified and marked. Core samples (1 mm diameter) were subsequently removed from each tumor donor block and re-embedded in a new recipient paraffin TMA block using a TMA Master Arrayer (3DHISTECH, Budapest, Hungary). If sufficient material was available, representative tumor (*n* = 3) and marginal tissue (*n* = 1) cores were sampled. Liver and placental cores were included in each TMA to facilitate orientation within the TMA during microscopy.

### HIF-1α assay

Immunohistochemistry (IHC) stains were performed on 3 µm TMA tissue sections according to standard protocols. Slides were stained using the Envision Flex + system (Agilent). Slides went through deparaffinization and epitope retrieval in a PT link with a low pH buffer (PT Link, Agilent, Santa Clara, CA 95051, USA). Next, epitope retrieval staining was carried out on an Autostainer Link 48 (Agilent). Endogenous enzyme activity was blocked for 10 min using EnVision FLEX Peroxidase-Blocking Reagent (Agilent, SM801). Sections were then incubated overnight 4 °C with the primary antibody HIF-1-α (clone EP1215Y) (abcam ab51608) in a 1:1000 dilution, followed by FLEX HRP secondary for 30 min (Agilent, SM802) and diaminobenzidine chromogene (DAB) (Agilent: EnVision FLEX DAB+ Chromogen (DM827) and EnVision FLEX Substrate Buffer (SM803)) for 10 min. Slides were counterstained using Hematoxylin (Agilent, EnVision FLEX Hematoxylin (K8008)) for 8 min. Slides were then mounted and scanned on a Hamamatzu Nanozoomer 2.0HT.

### TMA core scoring

Expression of HIF-1α was quantified with an H-score that incorporated staining intensity and percentage of positively stained tumor cells [18]. Staining intensity was a weighted scale ranging from 0 for no staining to 3 for high intensity staining. Percent positivity ranged from 0 to 100% based on percentage of positively stained tumor cells. In a set of sensitivity analyses, we used percent positivity to quantify HIF-1α expression.

**Fig. 2:**
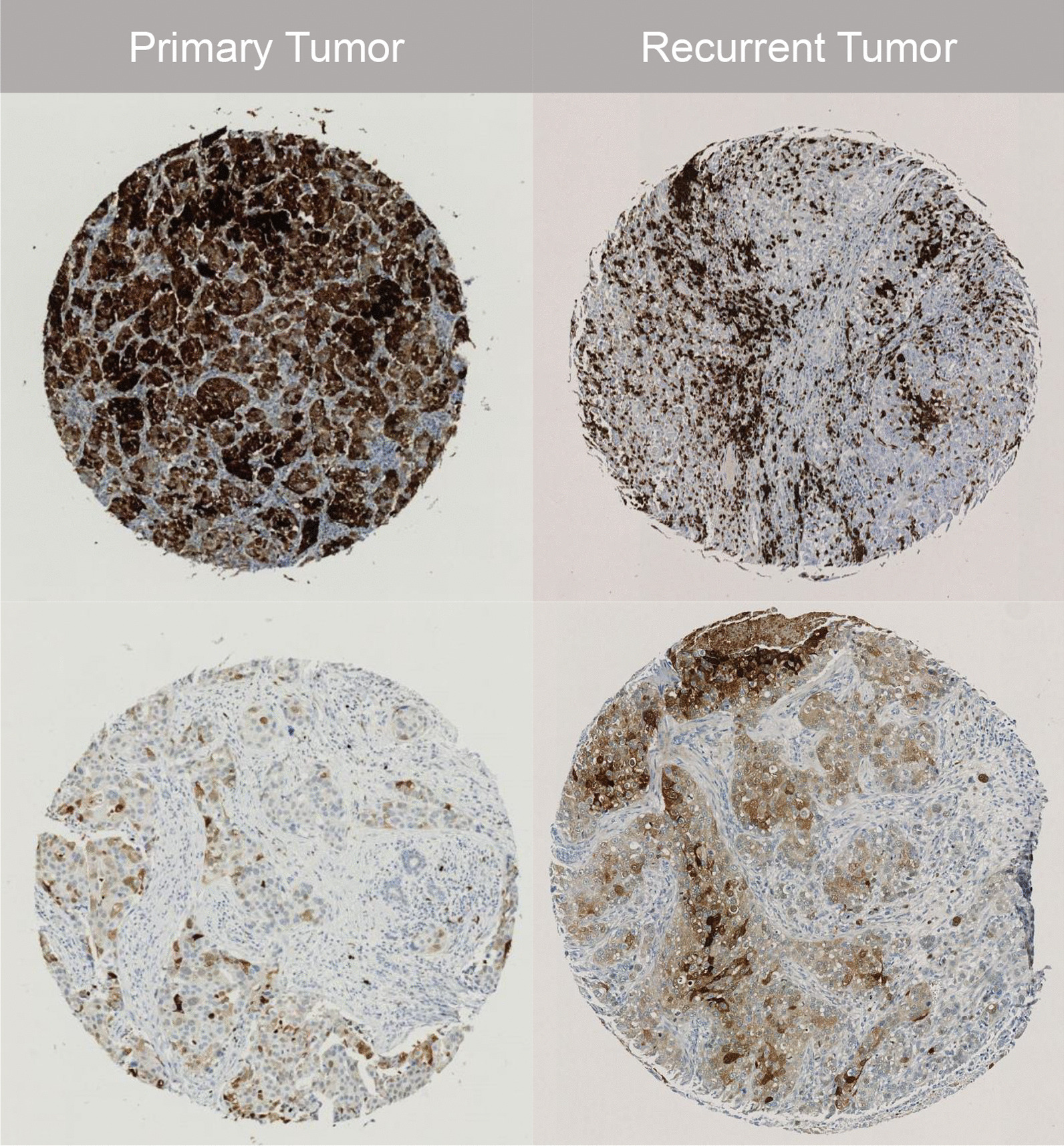
Immunohistochemical analysis of cytoplasmic HIF-1α in primary tumor cores and recurrent tumor cores

Two authors (LJC and SHD) developed a rubric for intensity levels and a scoring schematic of cytoplasmic HIF-1α expression (Fig. 2). HIF-1α levels in the cytoplasm are not transcriptionally active but are translocated to the nucleus where they exert biological activity. Levels of HIF-1α in the cytoplasm increase with response to hypoxia and therefore represent cancer cell adaptation to a hypoxic tumor microenvironment. Subsequently, all study cores were rated by one evaluator (LJC), who was blinded to patient characteristics and case–control status at the time of scoring. Cores that could not be scored were excluded, either because the core section on the TMA was absent or inadequately represented, or because of poor staining of the core, and this exclusion was addressed in the analysis. An average of cores available was used to assign a value of HIF-1α expression based on the average H-score and percent positivity for each patient.

### Analytic variables

#### Expression of HIF-1α

The exposure of interest for this study was cytoplasmic expression of HIF-1α, which we defined as positive versus negative (H-score > 10 as positive expression versus H-score ≤ 10 as negative expression). This cutpoint was based on the 90^th^ quantile of the distribution of the average H-score of HIF-1α expression among the controls (Additional file 1: Figure S1A and S1B). Among the cases, expression of HIF-1α in the paired recurrent tumors was compared with HIF-1α expression in the primary tumor. The difference in average H-score in the recurrent tumor and the average H-score in the primary tumor was used to determine if there was a decrease (difference in H-scores ≤  − 5), increase (difference in H-scores ≥ 5), or no change in HIF-1α expression (difference in H-scores between − 5 and 5).

### Breast cancer recurrence

The study followed the DBCG definition of breast cancer recurrence, *i.e.* any contralateral or ipsilateral breast cancer occurring locally, regionally, or distally, after breast cancer diagnosis. As noted above, all recurrences occurred one to ten years after initial breast cancer diagnosis. Time to recurrence was categorized by approximate quintiles among cases: 1 to < 2 years; 2 to < 3 years; 3 to < 4 years; 4 to < 6 years; and 6 to 10 years.

### Covariates

We included UICC stage (I, II, III), grade (I, II, III), menopausal status at diagnosis (premenopausal/postmenopausal), receipt of chemotherapy (yes/no), receipt of radiotherapy (yes/no), surgery type (mastectomy/breast conserving surgery), year of diagnosis, age at diagnosis, and county of residence in each analysis. During the study enrolment period, guidelines for TAM duration changed from one year to two years, and finally to five years of adjuvant therapy. Therefore, to account for the progression of duration in the guidelines, we adjusted for TAM treatment duration (years) in the ER+/TAM+ stratum.

### Statistical analysis

Analyses were stratified by the ER/TAM grouping to evaluate whether HIF-1α expression was predictive of tamoxifen resistance, prognostic of breast cancer recurrence, or neither predictive nor prognostic. We first report the covariate distributions as frequency and percent by case and control status within ER/TAM group. We additionally present descriptive statistics of ER/TAM group, HIF-1α expression in primary tumor, and time to recurrence by change in HIF-1α expression (decrease, no change, increase), comparing HIF-1α expression in the recurrent versus primary tumor.

In the conventional analysis, we used logistic regression to estimate the association between HIF-1α expression and breast cancer recurrence. To avoid discarding matched sets due to missing tumor core samples, we used unconditional multivariable logistic regression adjusting for the matched factors and other covariates to compute the ORs and 95% confidence intervals (CIs) reflecting the association of HIF-1α expression with recurrence.

To estimate the association between HIF-1α expression and time to recurrence, we calculated five ORs and the 95% CIs within the approximate quintiles of time to recurrence, adjusting for the same covariates included in the conventional analysis. We then regressed the natural logarithm of the ORs (lnOR) on time to recurrence (represented by *i* = 5 midpoints), weighting with the inverse variance of lnOR. The beta-estimate from this approach reflects the association between HIF-1α expression and time to recurrence.

In an exploratory analysis, we examined the role of conservation of HIF-1α expression—defined as positive expression in both the primary and recurrent tumor—and late recurrence. We calculated the OR associating conservation of HIF-1α expression with late recurrence (≥ 5 vs < 5 years) among cases with HIF-1α expression in the primary tumor.

Finally, we performed sensitivity analyses for the above set of analyses using percent positivity to define HIF-1α expression instead of the H-score.

### Quantitative bias analysis

There were some eligible patients for whom we were unable to collect FFPE blocks and some for whom we were unable to assay expression in the collected blocks (Additional file 1: Table S1). To account for potential baseline selection bias due to missing tumor cores (15% of ER+/TAM+ patients and 15% of ER−/TAM− patients), we performed a quantitative bias analysis. We used inverse probability of participant weighting (IPPW) to reweight the study population of women with complete expression data to the population we would have observed had all FFPE blocks been available (i.e., had all patients been included) [19]. Reweighting accounted for selection proportions within the matched factors and other potential confounders. We used bootstrapping with 500 samples to estimate the 95% confidence limits for the IPPW bias-adjusted ORs [20]. Analyses were performed using SAS 9.4 (Cary, NC) and R v3.6 (Vienna, Austria).

The study was approved by the Danish Data Protection Agency, the Danish Ethical Committee, and the Emory University Institutional Review Board.

## Results

In the ER+/TAM+ stratum, positive HIF-1α expression was observed in 23% of the cases and 20% of the controls, whereas in the ER−/TAM− stratum, we observed that 47% of cases and 48% of controls had positive HIF-1α expression (Table 1). The majority (96%) of participants were initially diagnosed with stage II or III breast cancer. Most women were postmenopausal at diagnosis (81%), although this differed across ER+/TAM+ and ER−/TAM− strata (93% vs. 60%, respectively). Among women diagnosed with ER+ disease, tamoxifen was prescribed for one year (47%) or five years (37%).

**Table 1:** Distribution of tumor and patient characteristics among breast cancer recurrence cases and controls by ER/TAM group among 1682 subjects from the ProBe CaRe population-based case control study

Patient characteristic	ER+/TAM+, N (%)		ER−/TAM−, N (%)			
	Case	Control	Case		Control	
*HIF-1α expression*						
Positive	106 (23)	93 (20)	122 (47)		122 (48)	
Negative	352 (77)	371 (80)	136 (53)		132 (52)	
Missing	83	77	42		46	
*Diagnosis year*						
1985–1993	235 (43)	234 (43)	107 (36)		100 (33)	
1994–1996	113 (21)	112 (30)	81 (27)		83 (28)	
1997–2001	193 (36)	195 (36)	112 (37)		117 (39)	
*Age category at diagnosis*						
35–44	16 (3.1)	13 (2.4)	68 (23)		58 (19)	
45–54	116 (21)	111 (21)	120 (40)		113 (38)	
55–64	286 (53)	281 (52)	82 (27)		86 (29)	
65–69	123 (23)	136 (25)	30 (10)		43 (14)	
*Menopausal status at diagnosis*						
Premenopausal	34 (6.3)	34 (6.3)	121 (40)		121 (40)	
Postmenopausal	507 (94)	507 (94)	179 (60)		176 (60)	
*UICC tumor stage at diagnosis*						
I	9 (1.7)	9 (1.7)	25 (8.3)		25 (8.3)	
II	250 (46)	250 (46)	153 (51)		153 (51)	
III	282 (52)	282 (52)	122 (41)		122 (41)	
*Histological grade*						
I	108 (25)	144 (35)	27 (11)		23 (10)	
II	234 (54)	215 (52)	125 (49)		98 (43)	
III	92 (21)	57 (14)	103 (40)		106 (47)	
Missing	107	125	45		73	
*Surgery type*						
Breast-conserving surgery	58 (11)	71 (13)	47 (16)		56 (19)	
Mastectomy	483 (89)	470 (87)	252 (84)		244 (81)	
*Radiation therapy*						
Yes	183 (34)	191 (35)	128 (44)		123 (47)	
No	358 (66)	350 (65)	166 (56)		137 (53)	
Missing			6		40	
*Systemic adjuvant chemotherapy*						
Yes	70 (13)	65 (12)	248 (83)		188 (63)	
No	471 (87)	476 (88)	52 (17)		112 (37)	
*Tamoxifen protocol, years*						
1	257 (48)	261 (48)				
2	98 (18)	92 (18)				
5	186 (34)	188 (34)				

ER, estrogen receptor; HIF**-**1α, hypoxia-inducible factor 1; TAM, tamoxifen; UICC, Union for International Cancer Control

Table 2 describes changes in HIF-1α expression between primary and recurrent tumor cores. Most (48%) had no meaningful change in HIF-1α expression, 23% had an increase, and 28% had a decrease in HIF-1α expression. Women in the ER+/TAM+ stratum were less likely to have a decrease in HIF-1α expression compared with women in the ER−/TAM− stratum (25% vs 33%). Among cases with recurrent tissue available, 61% of cases with positive HIF-1α expression in the primary tumor had no expression in the recurrent tumor. Conversely, among those with no HIF-1α expression in the primary tumor, 19% had positive HIF-1α expression in the recurrent tumor.

**Table 2:** Change in HIF-1α expression between primary tumor and recurrent tumor by ER/TAM group and time to recurrence among 269 recurrences from the ProBe CaRe population-based case control study

Characteristics	Change in HIF-1α expression^a^					
	Decrease		No change		Increase	
	*n*	%	*n*	%	*n*	%
Total	76	(28)	130	(48)	63	(23)
Median change [IQR]	− 18.2 [− 44.2, − 8.0]		0 [− 1.3, 1.3]		21.3 [9.5, 54]	
*ER/TAM group*						
ER+/TAM+	41	(25)	84	(51)	38	(23)
ER−/TAM−	35	(33)	46	(43)	25	(24)
*HIF-1α category*						
Positive	63	(79)	2	(2.5)	15	(19)
Negative	13	(6.9)	128	(68)	48	(25)
*Time to recurrence, years*						
1 to < 2	30	(31)	43	(45)	23	(24)
2 to < 3	12	(21)	32	(56)	13	(23)
3 to < 4	7	(16)	26	(58)	12	(27)
4 to < 6	17	(37)	18	(39)	11	(24)
6–10	10	(40)	11	(44)	4	(16)

ER, estrogen receptor; HIF**-**1α, hypoxia-inducible factor 1; IQR, interquartile range; TAM, tamoxifen

In the ER+/TAM+ stratum, we observed a near-null association in the odds of breast cancer recurrence among women with tumors positive for HIF-1α expression compared with those without HIF-1α expression (OR = 1.21, 95% CI: 0.88, 1.67) (Table 3). Similarly, in the ER−/TAM− stratum, we observed a near-null association between HIF-1α expression and breast cancer recurrence (OR = 0.97, 95% CI 0.68, 1.39). Accounting for potential selection bias with IPPW due to missing tumor cores yielded little change in the estimates of association across both ER/TAM groups.

**Table 3:** Association between HIF-1α expression and breast cancer recurrence by ER/TAM group among 1682 subjects from the ProBe CaRe population-based case control study

HIF-1α expression	ER+/TAM+ breast cancer patients			ER−/TAM− breast cancer patients		
	Recurrent cases/ controls	Adj. OR (95% CI)^a^	IPPW OR (95%CI)	Recurrent cases/	Adj. OR (95% CI)^a^	IPPW OR (95%CI)
Positive	106/93	1.21 (0.88, 1.67)	1.19 (0.87, 1.63)	122/122	0.97 (0.68, 1.39)	0.91 (0.60, 1.36)
Negative	352/371	Reference	Reference	136/132	Reference	Reference

Adj., adjusted; CI, confidence interval; ER, estrogen receptor; HIF**-**1α, hypoxia-inducible factor 1; IPPW, inverse probability of participant weighting; OR, odds ratio; TAM, tamoxifen

^a^Adjusted for matching factors (menopausal status, surgery date, county of residence, stage, age category) and chemotherapy, radiation therapy, and tamoxifen duration (ER+ stratum only)

In Table 4 we report the association between HIF-1α expression and time to recurrence. Across both ER+/TAM+ and ER−/TAM− strata, HIF-1α expression was associated with recurrence in years 3 to < 4 (OR = 3.41, 95% CI 1.28, 9.06 and OR = 2.50, 95% CI 0.81, 7.68, respectively), although the estimates were imprecise. However, no association was observed in other categories of time to recurrence, and there was no discernable pattern in the association between HIF-1α expression and breast cancer recurrence by category of time to recurrence. When we regressed the lnOR on time to recurrence, we observed no association in the ER+/TAM+ group (*β* = 0.005, 95% CI − 0.23, 0.24), but a negative association in the ER−/TAM− group (β =  − 0.27, 95% CI − 0.62, 0.08), suggesting that HIF-1α expression was associated with early recurrence in the ER−/TAM− group (Table 5).

**Table 4:** Association between HIF-1α expression and breast cancer recurrence by quintile of recurrence time and ER/TAM group among 1682 subjects from the ProBe CaRe population-based case control study

Time to recurrence	Median time to recurrence (yaers)	ER+/TAM+ breast cancer patients		ER−/TAM− breast cancer patients	
		Cases/controls	Adjusted OR (95% CI)^a^	Cases/controls	Adjusted OR 95% CI)^a^
1 to < 2	1.5	109/113	1.03 (0.54, 1.96)	113/105	0.73 (0.42, 1.27)
2 to < 3	2.4	80/85	1.16 (0.55, 2.44)	67/72	0.74 (0.36, 1.54)
3 to < 4	3.4	88/78	3.41 (1.28, 9.06)	32/33	2.50 (0.81, 7.68)
4 to < 6	4.7	113/115	0.79 (0.41, 1.52)	27/27	0.76 (0.22, 2.60)
6–10	7.3	68/73	1.39 (0.59, 3.30)	19/17	5.78 (0.61, 55.0)

CI, confidence interval; ER, estrogen receptor; OR, odds ratio; TAM, tamoxifen; y, years

^a^Adjusted for matching factors (menopausal status, surgery date, county of residence, stage, age category) and chemotherapy, radiation therapy, and tamoxifen duration (ER+ stratum only)

**Table 5:** Association between HIF-1a expression with time to recurrence by ER/TAM group among 1682 subjects from the ProBe CaRe population-based case control study

ER/TAM Group	Intercept (SE)	Effect estimate *β* (SE)	95% CI
ER+/TAM+	0.18 (0.50)	0.005 (0.12)	(− 0.23, 0.24)
ER−/TAM−	0.95 (0.51)	− 0.27 (0.18)	(− 0.62, 0.08)

CI, confidence interval; ER, estrogen receptor; SE, standard error; TAM, tamoxifen

In our exploratory analysis of the association between conservation of HIF-1α expression—positive HIF-1α expression in primary and recurrent tumors—and late recurrence, we observed an OR = 4.32 (95% CI 0.92, 20) for the association between conservation of HIF-1α expression and late breast cancer recurrence compared with loss of HIF-1α expression in the recurrent tumor in the ER+/TAM+ group. We were unable to calculate a reliable OR in the ER−/TAM− as there was only 1 case that had positive expression in both the primary and recurrent tumors, and a late recurrence.

The sensitivity analyses, which assessed HIF-1α positivity based on percent positive of tumor cells, yielded similar results to the analyses assessing HIF-1α expression based on the H-score (Additional file 1: Tables S2–S6).

## Discussion

In this study, we did not observe an association between HIF-1α expression and breast cancer recurrence or timing of breast cancer recurrence among women in the ER+/TAM+ stratum. However, we observed that HIF-1α expression may be associated with early recurrence among women in the ER−/TAM− stratum.

Previous studies have reported mixed associations between HIF-1α expression and breast cancer prognosis. A recent meta-analysis, which included 14 studies, reported that high HIF-1α expression among breast cancer patients was an indicator of poor prognosis, and was associated with both overall survival (hazard ratio [HR] = 1.46, 95%CI 1.12, 1.92) and disease-free survival (HR = 1.91, 95%CI 1.43, 2.57) [21]. However, studies included in the meta-analysis were relatively small (< 750 patients) and were heterogeneous with respect to positive HIF-1α expression classification. Dales et al. reported that overexpression of HIF-1α was associated with early recurrence among breast cancer patients, but ER status was not recorded for study participants [8]. The results reported by Dales et al. are consistent with our results among ER−/TAM− breast cancer patients. Another study examined the association between HIF-1α expression and recurrence-free survival in a cohort of premenopausal breast cancer patients from a randomized trial of TAM therapy [22]. The authors concluded that HIF-1α expression was associated with recurrence among those who did not receive TAM (HR = 1.4, 95% CI 0.9, 2.3).

Tumor hypoxia is an adaptive mechanism by which tumor cells are able to survive in the oxygen deprived tumor microenvironment, supporting cancer cell progression [5]. HIF-1α is frequently activated in tumors, which decreases hypoxia-induced apoptosis and increases stress-induced proliferation of solid tumors [23]. Some studies have reported that ER expression is inversely associated with HIF-1α expression [7, 24]. These reports are consistent with those observed in the current study, as women in the ER−/TAM− group were more likely to have positive HIF-1α expression. Moreover, we observed that HIF-1α expression was associated with an early time to recurrence among those in the ER−/TAM− group. Our exploratory analyses suggested that breast cancer patients in the ER−/TAM− group who had positive HIF-1α expression in their primary and recurrent tumors were more likely to have had early recurrence (< 5 years), although the small sample size yielded imprecise results.

A limitation of this study is that it was restricted to breast cancer diagnoses between 1985 and 2001. During this period, TAM represented guideline-concordant care for postmenopausal women, and for premenopausal women beginning in 1999, but is now frontline adjuvant hormone therapy only for premenopausal women. However, postmenopausal women for whom aromatase inhibitors are contraindicated or poorly tolerated still receive TAM; so our results are still applicable to that target population [25]. Although screening protocols were being developed during this time period, Denmark did not implement a nationwide breast cancer screening program until 2007, after this study’s enrollment period [26]. An additional concern is that the study population consisted primarily of women initially diagnosed with stage II (48%) and stage III (48%) disease, largely due to DBCG criteria for TAM therapy during the study’s diagnostic period [27]. However, TAM has for some time been prescribed to women with stage I ER+ breast cancer as guideline-concordant therapy. We were unable to assess recurrence risk after 10 years of follow-up, as the DBCG follows breast cancer patients for a recurrence for up to 10 years. Recurrences that occur after 10 years may be of interest for future studies. It is possible that we imperfectly measured HIF-1α expression in this study. We used the average of 1–3 cores per patient to mitigate any variation in scoring or intratumor heterogeneity and evaluated HIF-1α expression using both an average H-score and average percent positivity with consistent results. Finally, we only had recurrent tumors for a subset of the recurrent cases (32%). This is expected as women diagnosed with a distant recurrence are unlikely to have primary surgery as part of their care, but it limited our ability to estimate the association between changes in HIF-1α expression and timing of recurrence.

## Conclusions

In conclusion, in this population-based case–control study, we found no evidence of an association between HIF-1α expression and breast cancer recurrence, or timing of recurrence among ER+/TAM+ postmenopausal breast cancer patients. We observed some evidence of an association between HIF-1α expression and early recurrence among ER−/TAM− breast cancer patients. Future studies may be strengthened by examination of HIF-1α expression in premenopausal breast cancer patients and conservation of HIF-1α expression in tumors over time.

## Supplementary Information

**Additional file 1:** Supplementary materials for distribution of HIF-1α, availability of tumor cores, and analyses using percent positivity of HIF-1α.

## Data Availability

The data that support the findings of this study are available upon request to the corresponding author and the Department of Clinical Epidemiology. The data are not publicly available due to privacy and ethical considerations.

## Abbreviations

HIF-1α: Hypoxia-inducible factor-1α
ER: Estrogen receptor
TAM: Tamoxifen
OR: Odds ratio
CI: Confidence interval
DBCG: Danish Breast Cancer Group
UICC: Union for International Cancer Control
CPR: Civil Personal Registration
FFPE: Formalin-fixed, paraffin-embedded
TMA: Tissue microarray
IHC: Immunohistochemistry
IPPW: Inverse probability of participant weighting
HR: Hazard ratio

## Declarations

### Novelty and impact

Hypoxia-inducible factor-1α (HIF-1α) facilitates adaption of cancer cells to hypoxic tumor microenvironments and may be prognostic of breast cancer recurrence. In this study, we evaluated the association of HIF-1α expression with breast cancer recurrence and timing of recurrence in a population-based case–control study. We observed a near-null association between HIF-1α expression and breast cancer recurrence overall, and that HIF-1α expression may be associated with early recurrence among women diagnosed with estrogen receptor negative breast cancer.
