# Supplementary material for: Hypoxia-inducible factor-1α expression and breast cancer recurrence in a Danish population-based case control study
Source: Breast Cancer Res. 2021 Nov 4;23:103. doi: 10.1186/s13058-021-01480-1 (PMC8567651; doi:10.1186/s13058-021-01480-1)
Supplement: Supplementary file 1 — Additional file 1: Supplementary materials for distribution of HIF-1α, availability of tumor cores, and analyses using percent positivity of HIF-1α. [file 13058_2021_1480_MOESM1_ESM.docx]

**Supplemental Materials**

**Title**: Hypoxia-inducible factor-1α expression and breast cancer recurrence in a Danish population-based case control study

**Author List:** Lindsay J. Collin,^1,2,3^ Maret Maliniak,^1^ Deirdre P. Cronin-Fenton,^2^ Thomas P. Ahern,^4^ Kristina B. Christensen,^5^ Per Damkier,^6,7^ Stephen Hamilton-Dutoit,^5^ Kristina L. Lauridsen,^5^ Rami Yacoub,^1^ Peer Christiansen,^8^ Henrik Toft Sørensen,^2^ Timothy L. Lash^1^


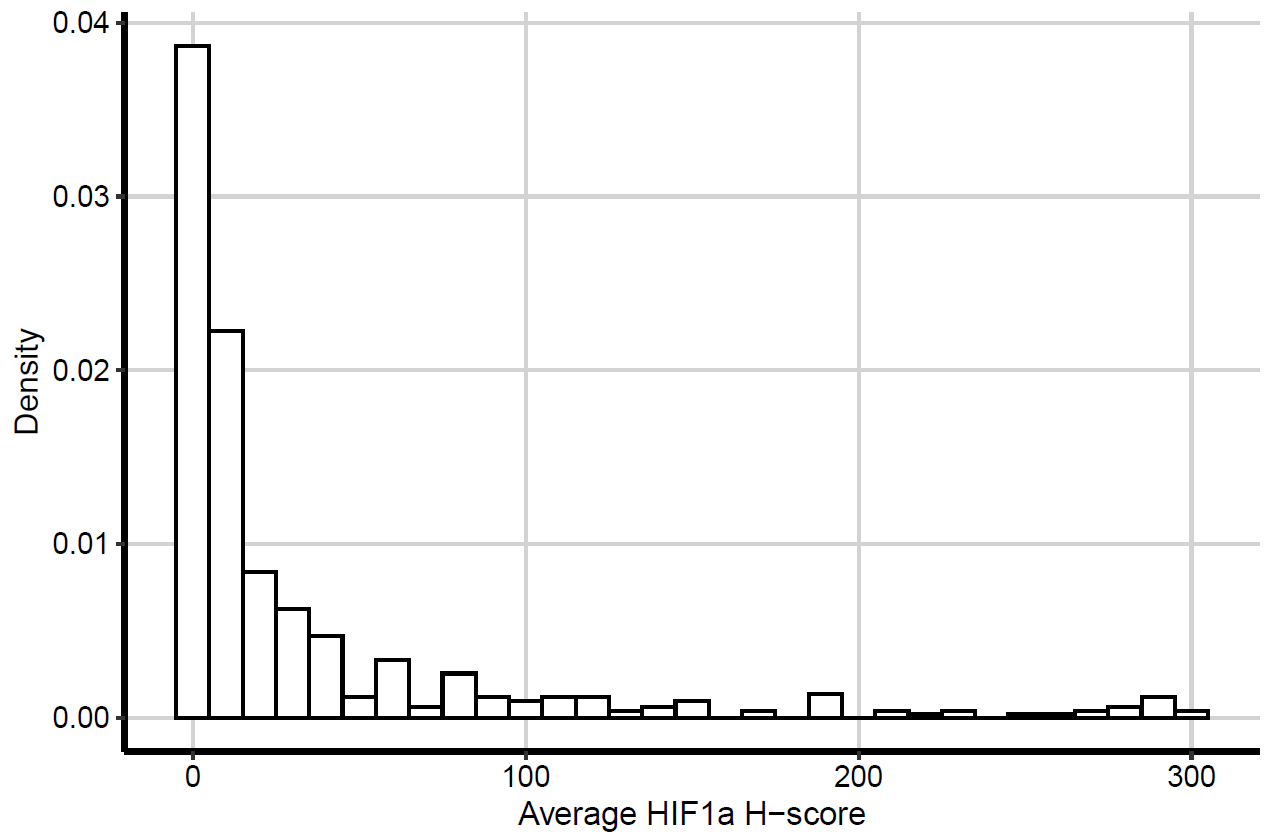
**Figure S1a and S1b:** Distribution of HIF-1α expression by A) ER+/TAM+ and B) ER−/TAM− groups among the 1682 breast cancer patients included in the ProBe CaRe population-based case control study in Denmark.

**B**A

**A**A


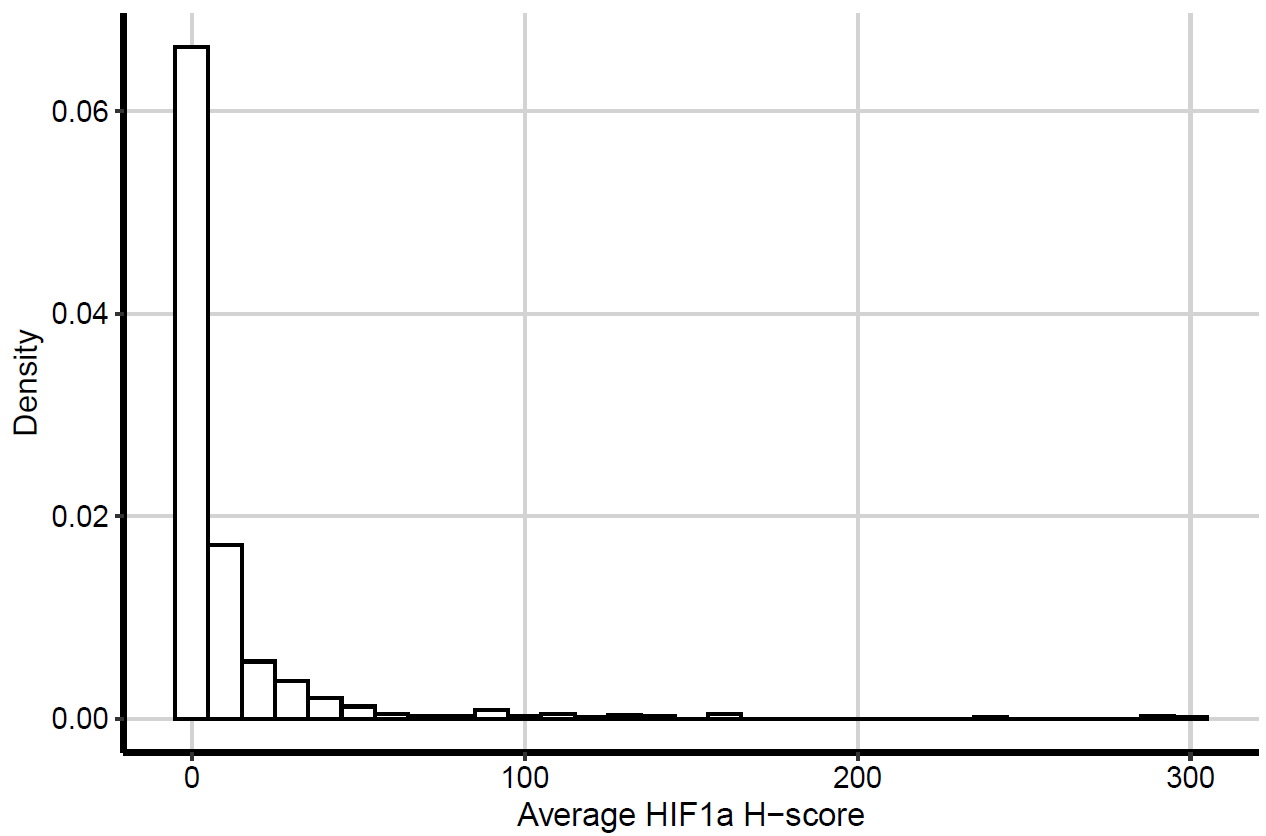


| **Table S1**: Distribution of tumor and patient characteristics among breast cancer recurrence cases and controls by tumor core availability and ER/TAM group among 1682 subjects from the ProBe CaRe population-based case control study. | | | | | | | | | | | | | | | | |
| --- | --- | --- | --- | --- | --- | --- | --- | --- | --- | --- | --- | --- | --- | --- | --- | --- |
| **Patient Characteristic** | **ER+/TAM+, N (%)** | | | | | | | | **ER−/TAM−, N (%)** | | | | | | | |
|  | **Case** | | | | **Control** | | | | **Case** | | | | **Control** | | | |
|  | **Tumor Core** | | **No Tumor Core** | | **Tumor Core** | | **No Tumor Core** | | **Tumor Core** | | **No Tumor Core** | | **Tumor Core** | | **No Tumor Core** | |
|  | **N** | **(%)** | **N** | **(%)** | **N** | **(%)** | **N** | **(%)** | **N** | **(%)** | **N** | **(%)** | **N** | **(%)** | **N** | **(%)** |
| **Total** | 458 | (85) | 83 | (15) | 464 | (86) | 77 | (14) | 258 | (86) | 42 | (14) | 254 | (85) | 46 | (15) |
| **Diagnosis year** |  |  |  |  |  |  |  |  |  |  |  |  |  |  |  |  |
| 1985–1993 | 194 | (83) | 41 | (17) | 195 | (83) | 39 | (17) | 90 | (84) | 17 | (16) | 81 | (81) | 19 | (19) |
| 1994–1996 | 92 | (81) | 21 | (19) | 96 | (86) | 16 | (14) | 67 | (83) | 14 | (17) | 73 | (88) | 10 | (12) |
| 1997–2001 | 172 | (89) | 21 | (11) | 173 | (89) | 22 | (11) | 101 | (90) | 11 | (9.8) | 100 | (85) | 17 | (15) |
| **Menopausal status at diagnosis** | |  |  |  |  |  |  |  |  |  |  |  |  |  |  |  |
| Premenopausal | 28 | (82) | 6 | (18) | 31 | (91) | 3 | (9) | 100 | (83) | 21 | (17) | 105 | (87) | 16 | (13) |
| Postmenopausal | 430 | (85) | 77 | (15) | 433 | (85) | 74 | (15) | 158 | (88) | 21 | (12) | 149 | (83) | 30 | (17) |
| **Age category at diagnosis** |  |  |  |  |  |  |  |  |  |  |  |  |  |  |  |  |
| 35–44 | 15 | (94) | 1 | (6) | 13 | (100) | 0 |  | 52 | (77) | 16 | (24) | 50 | (86) | 8 | (14) |
| 45–54 | 98 | (84) | 18 | (16) | 99 | (89) | 12 | (11) | 106 | (88) | 14 | (12) | 96 | (85) | 17 | (15) |
| 55–64 | 235 | (82) | 51 | (18) | 236 | (84) | 45 | (16) | 73 | (89) | 9 | (11) | 74 | (86) | 12 | (14) |
| 65–69 | 110 | (89) | 13 | (11) | 116 | (85) | 20 | (15) | 27 | (90) | 3 | (10) | 34 | (79) | 9 | (21) |
| **UICC tumor stage at diagnosis** | |  |  |  |  |  |  |  |  |  |  |  |  |  |  |  |
| I | 9 | (100) |  |  | 6 | (67) | 3 | (33) | 21 | (84) | 4 | (16) | 22 | (88) | 3 | (12) |
| II | 209 | (84) | 41 | (16) | 210 | (84) | 40 | (16) | 133 | (87) | 20 | (13) | 132 | (86) | 21 | (14) |
| III | 240 | (85) | 42 | (15) | 248 | (88) | 34 | (12) | 104 | (85) | 18 | (15) | 100 | (82) | 22 | (18) |
| **Histological grade** |  |  |  |  |  |  |  |  |  |  |  |  |  |  |  |  |
| I | 88 | (81) | 20 | (19) | 123 | (85) | 21 | (15) | 18 | (67) | 9 | (33) | 19 | (83) | 4 | (17) |
| II | 202 | (86) | 32 | (14) | 187 | (87) | 28 | (13) | 110 | (88) | 15 | (12) | 87 | (89) | 11 | (11) |
| III | 79 | (86) | 13 | (14) | 49 | (86) | 8 | (14) | 92 | (89) | 11 | (11) | 88 | (83) | 18 | (1.) |
| Missing | 89 | (83) | 18 | (17) | 105 | (84) | 20 | (16) | 38 | (84) | 7 | (16) | 60 | (82) | 13 | (18) |
| **Surgery Type** |  |  |  |  |  |  |  |  |  |  |  |  |  |  |  |  |
| Breast-conserving surgery | 46 | (79) | 12 | (21) | 61 | (86) | 10 | (14) | 38 | (81) | 9 | (19) | 50 | (89) | 6 | (11) |
| Mastectomy | 412 | (85) | 71 | (15) | 403 | (86) | 67 | (14) | 219 | (87) | 33 | (13) | 204 | (84) | 40 | (16) |
| **Radiation therapy** |  |  |  |  |  |  |  |  |  |  |  |  |  |  |  |  |
| Yes | 158 | (86) | 25 | (14) | 165 | (86) | 26 | (14) | 106 | (83) | 22 | (17) | 105 | (85) | 18 | (15) |
| No | 300 | (84) | 58 | (16) | 299 | (85) | 51 | (15) | 147 | (89) | 19 | (11) | 114 | (83) | 23 | (17) |
| Missing |  |  |  |  |  |  |  |  | 5 | (83) | 1 | (17) | 35 | (88) | 5 | (13) |
| **Systemic adjuvant chemotherapy** | |  |  |  |  |  |  |  |  |  |  |  |  |  |  |  |
| Yes | 60 | (86) | 10 | (14) | 53 | (82) | 12 | (18) | 218 | (88) | 30 | (12) | 157 | (84) | 31 | (17) |
| No | 398 | (85) | 73 | (15) | 411 | (86) | 65 | (14) | 40 | (77) | 12 | (23) | 97 | (87) | 15 | (13) |
| **Tamoxifen protocol, years** |  |  |  |  |  |  |  |  |  |  |  |  |  |  |  |  |
| 1 | 217 | (84) | 40 | (16) | 221 | (85) | 40 | (15) |  |  |  |  |  |  |  |  |
| 2 | 75 | (77) | 23 | (23) | 75 | (82) | 17 | (18) |  |  |  |  |  |  |  |  |
| 5 | 166 | (89) | 20 | (11) | 168 | (89) | 20 | (11) |  |  |  |  |  |  |  |  |

| **Table S2:** Change in HIF-1a expression (percent positivity) between primary tumor and recurrent tumor by ER/TAM group and time to recurrence by ER/TAM group among 269 recurrences from the ProBe CaRe population-based case control study. | | | | | | |
| --- | --- | --- | --- | --- | --- | --- |
| Characteristics | **Change in HIF-1α Expression** | | | | | |
|  | **Decrease** | | **No Change** | | **Increase** | |
| **Characteristics** | **n** | **%** | **n** | **%** | **n** | **%** |
| **Total** | 80 | (30) | 118 | (44) | 71 | (26) |
| Median [IQR] Change | −9.0 [−30.5, −4.7] | | 0, [−0.67, 0.67] | | 7.7 [4.0, 18.0] | |
| **ER/TAM Group** |  |  |  |  |  |  |
| ER+/TAM+ | 45 | (28) | 73 | (45) | 45 | (28) |
| ER−/TAM− | 35 | (33) | 45 | (42) | 26 | (25) |
| **HIF1 cat** |  |  |  |  |  |  |
| Positive | 45 | (83) | 0 |  | 10 | (18) |
| Negative | 35 | (16) | 118 | (55) | 61 | (29) |
| **Time to recurrence (years)** | |  |  |  |  |  |
| 1 to <2 | 32 | (33) | 41 | (43) | 23 | (24) |
| 2 to <3 | 13 | (23) | 30 | (53) | 14 | (25) |
| 3 to <4 | 10 | (22) | 18 | (40) | 17 | (38) |
| 4 to <6 | 17 | (37) | 17 | (37) | 12 | (26) |
| 6 to 10 | 8 | (32) | 12 | (48) | 5 | (20) |

Difference between recurrent and primary HIF-1α expression: Decrease= change < −2%; No change= −2% and 2%; Increase=change >2%

| **Table S3:** Association between HIF-1a expression (percent positivity) and breast cancer recurrence by ER/TAM group among 1682 subjects from the ProBe CaRe population-based case control study. | | | | | | |
| --- | --- | --- | --- | --- | --- | --- |
|  | **ER+/TAM+ breast cancer patients** | | | **ER−/TAM− breast cancer patients** | | |
| **HIF-1α Expression** | **Recurrent cases /controls** | **Adj. OR (95% CI)** | **IPPW OR (95%CI)** | **Recurrent cases/ controls** | **Adj. OR (95% CI)** | **IPPW OR (95%CI)** |
| Positive | 71/61 | 1.23 (0.84, 1.78) | 1.20 (0.84, 1.73) | 83/86 | 0.72 (0.48, 1.09) | 0.74 (0.48, 1.14) |
| Negative | 387/403 | Reference | Reference | 175/168 | Reference | Reference |

| **Table S4:** Association between HIF-1a expression (percent positivity) and breast cancer recurrence by quintile of recurrence time and ER/TAM group among 1682 subjects from the ProBe CaRe population-based case control study. | | | | | |
| --- | --- | --- | --- | --- | --- |
|  |  | **ER+/TAM+ breast cancer patients** | | **ER−/TAM− breast cancer patients** | |
| **Time to recurrence (years)** | **Median Time to Recurrence (y)** | **cases/ controls** | **Adjusted OR (95% CI)** | **cases/ controls** | **Adjusted OR (95% CI)** |
| 1 to <2 | 1.5 | 109/113 | 1.07 (0.51, 2.25) | 113/105 | 0.91 (0.51, 1.62) |
| 2 to <3 | 2.4 | 80/85 | 0.87 (0.35, 2.19) | 67/72 | 0.56 (0.26, 1.20) |
| 3 to <4 | 3.4 | 88/78 | 2.80 (0.92, 8.54) | 32/33 | 3.08 (0.77, 12.2) |
| 4 to <6 | 4.7 | 113/115 | 1.07 (0.51, 2.24) | 27/27 | 0.48 (0.14, 1.59) |
| 6 to 10 | 7.3 | 68/73 | 1.30 (0.49, 3.84) | 19/17 | 2.10 (0.35, 12.8) |

| **Table S5:** Association between HIF-1a expression (percent positivity) with time to recurrence by ER/TAM group among 1682 subjects from the ProBe CaRe population-based case control study. | | | | |
| --- | --- | --- | --- | --- |
| **ER/TAM Group** | **Intercept (SE)** | **Effect estimate (β), median time to recurrence (SE)** | | **95% CI around β** |
| ER+/TAM+ | 0.12 (0.39) | 0.02 (0.09) | (−0.16, 7.66) | |
| ER−/TAM− | 1.04 (0.49) | −0.32 (0.17) | (−0.65, 0.01) | |
